# A Rapid and Satisfactory Outcome with Combined Immunosuppressive Therapy in Acquired Haemophilia A with Underlying Tuberculosis

**DOI:** 10.1155/2022/2271228

**Published:** 2022-11-29

**Authors:** G. A. C. Gamakaranage, C. Kulathilake, P. G. N. Nadeeshani, K. H. B. P. Fernandopulle, C. S. Moonesinghe, D. Gunawardena, R. S. Gamage, N. Perera, J. Indrakumar

**Affiliations:** ^1^Department of Pathology, Faculty of Medical Sciences, University of Sri Jayewardenepura, Nugegoda, Sri Lanka; ^2^Professorial Medical Unit, Colombo South Teaching Hospital, Dehiwala-Mount Lavinia, Sri Lanka; ^3^Department of Medicine, Faculty of Medical Sciences, University of Sri Jayewardenepura, Nugegoda, Sri Lanka

## Abstract

Acquired haemophilia A (AHA) is a rare disorder with an incidence of 1.5 cases per million per year in the United Kingdom. The incidence could be underestimated due to difficulty in diagnosis and also due to the fact that people with low titre inhibitor levels are asymptomatic. It is usually a disease affecting elderly but a disease peak in the younger population is known. The common underlying diseases are autoimmune disorders, malignancies, infections, and drugs. However, approximately 50% of the cases do not have a specific aetiology and about 10% will not have bleeding manifestations. Therefore, an isolated prolongation of APTT should be evaluated, especially prior to any haemostatic challenges. We report a case of a middle-aged man who presented with bleeding due to AHA associated with high inhibitory titres and active pulmonary tuberculosis. He was treated with both antituberculous and combined-aggressive immunosuppressive therapy which resulted in satisfactory disease remission.

## 1. Introduction

Acquired haemophilia A (AHA) is a rare disorder with an incidence of 1.5 per million per year in the United Kingdom [1]. The incidence of these cases in Sri Lanka is not known. The number of reported cases could be underestimated due to difficulty in diagnosis and the fact that people with low titre inhibitor levels do not have bleeding manifestations. It is usually a disease affecting the elderly population. However, a peak incidence in the younger population is recognized. The main underlying aetiologies are autoimmune disorders, malignancies, infections, and drugs [1]. AHA can be very rarely seen during the initial postpartum period [1, 2]. Approximately 50% of the cases do not have a specific aetiology and about 10% will not have bleeding manifestations [1]. Therefore, an isolated prolongation of APTT should be evaluated, especially prior to any haemostatic challenges.

Acquired haemophilia occurs due to acquired coagulation inhibitors resulting in an immune-mediated depletion or inhibition of a coagulation factor, most commonly directed against coagulation factor VIII (FVIII) [1]. Unlike FVIII inhibitors formed in congenital haemophilia A (alloantibodies against factor VIII concentrates), acquired inhibitors (auto antibodies) show complex second order kinetics with incomplete inhibition which results in some level of residual FVIII activity, which can be detected in the laboratory but does not correlate with severity of clinical bleeding [3]. AHA has a significant mortality of 8%–42%, and 3–12% of deaths are caused by effects of immunosuppression resulting in infection and 3–8% due to bleeding [1].

Bruising, retroperitoneal, muscle, gastrointestinal, and urogenital bleeding are common in AHA, but haemarthrosis is uncommon in contrast to congenital haemophilia A [1]. The management of AHA includes effective control of bleeding manifestations, elimination of the inhibitor, and treatment of any underlying aetiology to cure the disease. As such, AHA requires an individualized therapeutic approach [1].

## 2. Case History

A 46 year old man presented with a 7 month history of episodic pain and swelling of left knee joint, nocturnal fever, productive cough, loss of appetite, and on and off painful erythematous skin nodules on arms and legs. He was a known patient with type 2 diabetes mellitus who was not receiving proper treatment. He was a smoker and admitted to excessive alcohol consumption. He was not taking any long-term medication. On examination, he was averagely built with a swollen, warm, and tender left knee joint with restricted movements. There were tender subcutaneous nodules over the left arm and right thigh, compatible with a clinical diagnosis of erythema nodosum. He also had finger clubbing and bilateral cervical and axillary lymphadenopathy but no hepatosplenomegaly or organomegaly, and the rest of the examination was essentially normal.

His initial investigations revealed a normochromic normocytic anaemia with haemoglobin of 84 g/L, moderate rouleaux formation, normal platelet, and white cell count with neutrophil predominance. The blood picture showed evidence of an infective or inflammatory process. His inflammatory markers were elevated, with a CRP of 42 mg/L and an erythrocyte sedimentation rate (ESR) of 138 mm/1^st^ hour.

Renal and liver profiles were normal apart from hypoalbuminaemia with reversed albumin globulin ratio. Lactate dehydrogenase level (LDH) was elevated (398.4 U/L). His chest radiograph revealed prominent bilateral hilar lymphadenopathy. Contrast-enhanced computed tomography (CECT) of the abdomen and chest revealed multiple discrete lymphadenopathy in the bilateral axillae, cervical, hilar, paratracheal, subcarinal, and para-aortic regions. Additionally, the high-resolution computed tomography chest revealed paraseptal emphysema, fibrosis, a thick-walled cavity in the right upper lobe apical segment with a ground glass appearance, and a patchy consolidation in the left lingual region. Findings were more suggestive of an infective pathology than sarcoidosis. Mantoux test was 13 mm and sputum was thrice negative for acid fast bacilli initially. At this point, the patient defaulted on follow-up for 3 months until he presented to the medical casualty with severe pain and swelling of the left knee joint and painful subcutaneous nodules on the legs and arms. The joint aspirate was blood stained and subsequently diagnosed as haemarthrosis. A skin biopsy was done for the skin nodules as well, for which he developed delayed bleeding from the biopsy site.

A coagulation screen was performed, which revealed an isolated prolongation of activated partial thromboplastin time (APTT) of 139 seconds with normal prothrombin time, thrombin time, and fibrinogen levels. Further inquiry revealed no family history of bleeding tendency, but his episodic left knee joint swelling over the past few months was suggestive of recurrent haemarthrosis.

A detailed coagulation work-up was done at this time. APTT mixing test with pooled normal plasma (PNP) revealed uncorrected APTT and that the FVIII and factor IX levels were markedly low at 0.3% and 0.006%, respectively. Factor XI was undetectable. The inhibitor assay showed a very high titre of FVIII inhibitor of >1000 Bethesda units (BU).

A diagnosis of AHA was made, and a possible aetiology was sought. An autoimmune screen revealed weakly positive antinuclear antibodies, but double-stranded DNA and rheumatoid factor were negative.

With a strong suspicion of active tuberculosis, the CECT chest and abdomen were repeated which revealed a cavity with a tree in bud appearance in the right upper lobe with a progressive right hilar lymphadenopathy suggestive of active pulmonary tuberculosis with bronchogenic spread. His fourth and fifth sputum samples were positive for acid-fast bacilli which confirmed pulmonary tuberculosis. An APL antibody screen was done which revealed weakly positive DRVVT, moderately positive antibeta 2 glycoprotein IgM (53.8 U/L), and a weakly positive anticardiolipin IgM levels. Repeat testing after 3-months confirmed the persistence of APL antibodies with a moderately positive DRVVT.

A final diagnosis of AHA due to a possible underlying autoimmune disease in the background of active pulmonary tuberculosis was made. It was not clear whether pulmonary tuberculosis contributed to autoimmunity. However, the coexistence of secondary APS was still considered due to the persistence of APL antibodies.

Patient had several bleeding episodes involving the left knee joint and the right elbow joint. He also developed a gluteal muscle haematoma and all bleeding manifestations were successfully managed with recombinant factor VIIa (r FVIIa) infusions and tranexamic acid. Following a multidisciplinary meeting, antituberculous drugs (ATD) for pulmonary tuberculosis were started. Concurrent immunosuppression therapy commenced with high dose steroids including initial intravenous methyl prednisolone followed by oral prednisolone. After a period of 10 weeks, oral cyclophosphamide 100 mg daily was added while tapering off oral steroids as the APTT was persistently prolonged with poor response. With cyclophosphamide, APTT was reduced to 50 s and the inhibitor titre came down to 700 BU. He responded well to ATD, with clinical improvement, a declining ESR, and clearance of the initial findings on his repeat CECTs.

Unfortunately, the patient developed transaminitis during this time of high-dose cyclophosphamide therapy and ATD therapy requiring drug withdrawal. Interruption of cyclophosphamide therapy resulted in several episodes of bleeding (haemarthrosis and epistaxis) which required aggressive factor replacement therapy. As he had almost completed 11 months of therapy, ATD was also discontinued at the same time.

At this point, a combined-aggressive immunosuppression therapy was started with rituximab, high-dose oral prednisolone and low-dose cyclophosphamide, with close monitoring of liver functions aimed at disease remission.

He showed a dramatic response just after the third dose of rituximab with normalization of APTT with both lupus sensitive and insensitive reagents and normalization of ESR after completion of rituximab 4 doses.

He was given low-dose (50 mg daily) therapy with cyclophosphamide which was continued for another 6 months, and prednisolone was tailed off slowly with monthly monitoring of APTT, ESR, and liver functions.

Subsequent regular assessments showed persistently normal APTT with both lupus-sensitive and insensitive reagents and normalized ESR with a normal FVIII level (70%) and factor IX (90%). But we could not perform the Factor XI levels due to resource constraints at that time.

A repeat APL screen with DRVVT was still positive which confirmed the persistence of APL antibodies. We considered this could more likely to be due to secondary APS with an underlying, undetermined autoimmune disorder than tuberculosis which had been completely treated by that time.

## 3. Discussion

We present a complicated case of AHA with concurrent active tuberculosis and the persistent presence of APL antibodies. We suspected that these autoantibodies could be due to tuberculosis or an underlying autoimmune disorder.

The occurrence of autoantibodies in active tuberculosis has been described. A randomized case-control study done in Taiwan found that a significant proportion of patients (32%) with active TB in endemic areas have elevated autoantibody titres, especially anticardiolipin IgG and anti-Scl70 which do not associate with the typical manifestations of the particular autoimmune disease. As these autoantibodies decrease or normalize in titres with the control of TB, it is suggested that they do not require any immunosuppressant therapy [4]. There are reported cases of AHA in patients with tuberculosis in the literature where the acquired inhibitor has been successfully eradiated with ATD alone [4, 5, 9, 10]. With the presence of an abnormally high titre of inhibitory levels with a recurrent bleeding in this patient, he required strong combined immunosuppression therapy from the beginning [12].

An unusually high titre of inhibitor of FVIII detected in the Bethesda assay and a very low FVIII level despite no spontaneous bleeding initially could be due to the assay interference of these APL antibodies. This could have been overcome if we used a lupus insensitive reagent to perform the Bethesda assay and a lupus insensitive reagent or a chromogenic assay to do the factor assay. The presence of active TB and uncontrolled diabetes were significant concerns to start strong immunosuppressive therapy in our patient initially. However, regardless of many challenges such as interrupted immunosuppressive therapy due to hepatotoxicity, the continuation of uninterrupted ATD for 11 months cured his TB. But the inhibitory status and the APL antibodies persisted, indicating that autoimmunity was probably not due to tuberculosis.

He was a good candidate for combined immunosuppressive therapy with rituximab, as he had a very high inhibitory titre [12, 13]. With the completion of 4 doses of rituximab, he showed a good response having a normalized APTT with both lupus sensitive and insensitive reagents and a good clinical improvement as well.

We did not consider thromboprophylaxis for him considering the presence of APL antibodies during remission due to several reasons. His FVIII level was not high initially (70%), and he was not compliant in taking anticoagulants. He will be further assessed with a repeat factor assay and DRVVT along with risk assessment for thrombosis during follow-up.

## 4. Conclusion

Even though AHA is a rare disease, it is challenging to manage affected patients who have high mortality and morbidity. Management is further difficult in a low-resource country as AHA requires costly haemostatic therapy and laboratory investigations.

Prompt evaluation, diagnosis, effective timely management of bleeding episodes, and appropriate individualized therapy to eradicate the inhibitor are essential to achieve a good outcome. This case needed a multidisciplinary approach with the involvement of a haematologist, physician, respiratory specialist, radiologist, and laboratory expertise for timely decision-making and to deliver an effective and comprehensive treatment for a successful story.

The dramatic response seen with combined immunosuppressive therapy with rituximab highlights that prompt and appropriate therapy is lifesaving in this rare disease.

## Data Availability

No data were used to support this study.

## Abbreviations

AHA: Acquired haemophilia A
APTT: Activated partial thromboplastin time
ATD: Antituberculous drugs
BU: Bethesda unit
CECT: Contrast-enhanced computed tomogram
HRCT: High-resolution computed tomogram
MDT: Multidisciplinary team.
